# Endometriosis: An Unusual Cause of Bilateral Pneumothoraces

**DOI:** 10.5811/cpcem.2019.11.45061

**Published:** 2020-01-24

**Authors:** Christopher S. Sampson, Kathleen White

**Affiliations:** * University of Missouri-Columbia, Department of Emergency Medicine, Columbia, Missouri; † University of Missouri-Columbia, Department of Surgery, Columbia, Missouri

## Abstract

A 27-year-old female presented to the emergency department with sudden onset shortness of breath. A diagnosis of bilateral catamenial pneumothoraces was made following chest radiograph. Catamenial pneumothorax is a recurrent spontaneous pneumothorax that occurs in 90% of affected women 24–48 hours after the onset of their menstruation; 30–50% of cases have associated pelvic endometriosis. Symptoms can be as simple as chest pain or as severe as the presentation of this patient who was initially found to be in significant respiratory distress.

## INTRODUCTION

Catamenial pneumothorax is a recurrent spontaneous pneumothorax that occurs in 90% of affected women 24–48 hours after the onset of their menstruation. Thirty to fifty percent of cases have associated pelvic endometriosis. Symptoms can be as simple as chest pain or as severe as the symptoms with which this patient presented.^1^ The number of known episodes of catamenial pneumothorax prior to treatment average between two and eight.^2^ We describe a rare presentation of catamenial pneumothorax given the bilateral presentation.

## CASE REPORT

**Image 1:**
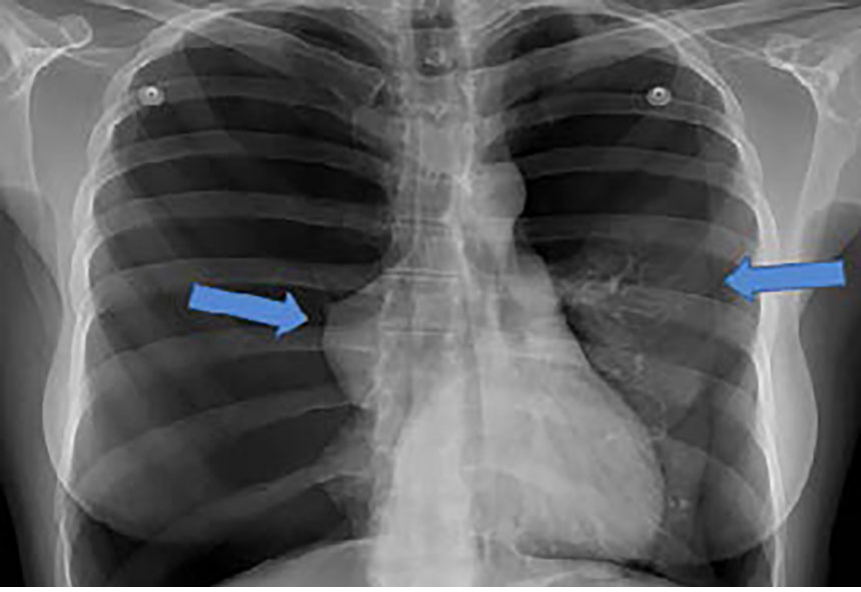
Posterior-anterior chest radiograph with bilateral pneumothoraces. Lung marking indicated by arrows.

A 27-year-old female presented to the emergency department (ED) by emergency medical services with a complaint of sudden-onset shortness of breath. She was working as a housekeeper when she had a sudden onset of severe chest pain and dyspnea. She reported being unable to take deep breaths since symptom onset. The patient had similar episodes in the past with a negative workup when she saw her doctor. Vital signs were notable for a respiratory rate of 28 breaths per minute and a pulse oximetry 95% on room air. Pulse rate and blood pressure were normal. On presentation, the patient was bent over in a chair in obvious distress. Lung sounds were diminished bilaterally when examined in a noisy hallway. A chest radiograph (CXR) was obtained (Image 1). Following CXR review, the patient was moved to a stretcher and placed on 100% oxygen via non-rebreather mask.

**Image 2:**
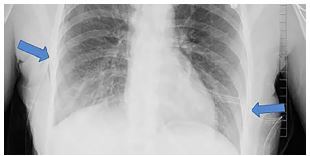
Chest radiograph following bilateral chest tube placement for bilateral pneumothoraces. Arrows mark chest tube insertion sites.

Upon further questioning a history of endometriosis was obtained along with a temporal relationship noted between her previous symptoms and her menstrual cycle. She was diagnosed with bilateral catamenial pneumothoraces, and bilateral chest tubes were placed without difficulty (Image 2). The patient was admitted to the medical inpatient service with pulmonary consultation. Thoracic endometriosis was confirmed following video-assisted thoracic surgery (VATS) with talc pleurodesis on hospital day nine.

## DISCUSSION

Catamenial pneumothorax (CP) is a rare disease with a poorly understood etiology that is commonly misdiagnosed as a spontaneous pneumothorax. Theories exist to explain its development but without any consensus. The disease is characterized by recurrent spontaneous pneumothorax that occurs 24 hours preceding or 72 hours following the onset of menses.^3^,^4^ Thus, it should be considered in the differential diagnosis of any menstruating female with recurrent pneumothorax.

CP was first characterized in the literature in 1958 by Maurer et al. as a recurrent, right-sided pneumothorax related to menstruation.^5^ In 1972 Lillington et al. termed the condition catamenial pneumothorax. Their report described a set of common clinical features among the cases including right-sided pneumothorax, close temporal relationship with onset of menses, and later onset closer to the fourth decade of life.^6^ In 2011 Haga et al. created a scoring system to help better distinguish CP from a spontaneous pneumothorax. This system included four clinical variables: side of pneumothorax; history of pelvic endometriosis; patient age; and smoking history. Right-sided pneumothorax had the highest odds ratio for CP.^7^

Previous literature has suggested that 3–6% of spontaneous pneumothoraces in women met the definition of CP; however, more recent studies have suggested that the actual rate is much higher and could be as high as 35%, although many of these studies were done on patients undergoing surgery.^4^, ^7^–^9^ The vast majority of cases of CP described in the literature have been unilateral with an estimated 85–95% being exclusively right sided. Left-sided CP is possible but rare, with bilateral CP being even more rare.^9^

Given the patient’s reported previous symptoms in relation to her menstrual cycle, it would be possible she had recurrent small pneumothoraces that spontaneously resolved. Her workup also occurred in the outpatient setting, and it was unknown whether any imaging had ever been obtained prior to the ED visit. VATS is the preferred procedure in these patients with attention not only given to lungs and thoracic cavity, but to the diaphragm as well. The diaphragm can develop defects or perforations from damage caused by the endometrial tissue.^11^

## CONCLUSION

Although catamenial pneumothorax is a rare cause of spontaneous pneumothorax in women, it should be an important consideration. Often with good history taking, including temporal relation to menstrual cycle, the diagnosis may become more apparent. Initial management is similar to any other pneumothorax, but may include surgical management to prevent recurrence.

**CPC-EM Capsule**

What do we already know about this clinical entity?

Endometriosis is a rare cause of pneumothorax in young women when there is pulmonary and diaphragmatic involvement.

What makes this presentation of disease reportable?

Bilateral catamenial pneumothoraces is an extremely unusual presentation of this disease.

What is the major learning point?

In any menstruating female with recurrent chest pain that is temporally related to her menstrual cycle who develops a pneumothorax, consider endometriosis as a cause.

How might this improve emergency medicine practice?

Recognition of catamenial pneumothorax can help patients obtain appropriate consultation for definitive care.
